# Single day 14 serum hCG values allow prediction of viable pregnancy and are significantly higher in frozen as compared to fresh single blastocyst transfer

**DOI:** 10.1007/s10815-024-03164-z

**Published:** 2024-06-13

**Authors:** Philip Sebastian Trautner, Peter Oppelt, Sarah Resch, Simon Hermann Enzelsberger, Thomas Ebner, Omar Josef Shebl

**Affiliations:** Department of Gynecology, Obstetrics and Gynecological Endocrinology, Kepler University Hospital, Johannes Kepler University Linz, Altenbergerstrasse 69, 4040 Linz and Krankenhausstrasse 26-30, 4020 Linz, Austria

**Keywords:** Human chorionic gonadotropin, Pregnancy monitoring, IVF, Threshold values, Embryo transfer, Predictive value

## Abstract

**Purpose:**

To evaluate if single serum human chorionic gonadotropin (hCG) level measurements are sufficient for pregnancy monitoring after single embryo transfer (sET) and to compare the hCG levels between fresh (FRET) and frozen embryo transfers (FET) in medically assisted reproduction.

**Methods:**

This was a retrospective exploratory cohort study including all patients who met the inclusion criteria, who received a single FRET (*n* = 249) or FET (*n* = 410) of a day five blastocyst at the IVF clinic at the Johannes Kepler University Linz between 2011 and 2020. hCG levels were measured on day 14 after embryo transfer. Threshold values for the viability of pregnancies were determined using receiver operating characteristic (ROC) curves.

**Results:**

Significantly higher hCG levels were found in those who received FET than in those who received FRET (1222.8 ± 946.7 mU/ml vs. 862.7 ± 572.9 mU/ml; *p* < 0.001). Optimal threshold values predicting a viable pregnancy were 368.5 mU/ml and 523 mU/ml in the FRET and FET groups, respectively.

**Conclusions:**

After FET, higher hCG values after 14 days of embryo transfer must be considered in pregnancy monitoring. Additionally, a single threshold hCG value seems to be sufficient for determining pregnancy viability. To exclude ectopic pregnancies, subsequent ultrasound examination is a mandatory requirement.

**Supplementary Information:**

The online version contains supplementary material available at 10.1007/s10815-024-03164-z.

## Introduction

In 1927, scientists demonstrated the presence of human chorionic gonadotropin (hCG), a gonad-stimulating substance, in the blood and urine of pregnant women for the first time [1]. Antibody-dependent pregnancy tests were first developed in the 1960s; however, it took two more decades for it to become commercially available as home-based pregnancy tests [2].

HCG consists of two subunits: the unspecific alpha subunit (92 amino acids), which also forms a part of other glycopeptide hormones such as thyroid-stimulating hormone (TSH), follicle-stimulating hormone (FSH), or luteinizing hormone (LH), and the specific beta subunit with 145 amino acids [3]. After nidation, hCG is produced rapidly by the syncytiotrophoblasts surrounding the embryo until it reaches a peak at the 10th week of gestation. HCG has several functions. It is responsible for the high progesterone production from the corpus luteum in the first weeks of natural pregnancy and for the differentiation of cytotrophoblasts to multinucleated syncytiotrophoblasts [4].

The use of medically assisted reproduction (MAR) has increased worldwide over the last few years, and this trend continues to increase. During this procedure, clinicians choose either fresh embryo transfer (FRET) or frozen embryo transfer (FET) with different pre-treatments for each method. After embryo transfer (ET), serum hCG level estimation is widely used for monitoring pregnancy and outcomes; it is usually first evaluated 9–17 days after ET. However, to date, there are no recommendations on which day or the number of times hCG levels should be estimated.

We aimed to test whether there was a difference between hCG values taken 14 days after single fresh embryo transfer (sFRET) and single frozen embryo transfer (sFET), and to determine the threshold values for the viability of the resulting pregnancies.

## Material and methods

This was a retrospective exploratory cohort study analysing data of single blastocysts transfers which were performed between 2011 and 2020. The patients were divided into two groups: Group A, those undergoing FRET and Group B, those undergoing FET. In order to select fresh and vitrified/warmed blastocysts for transfer Gardner’s criteria were applied [5]. In short, these criteria are based on three parameters, the overall expansion of the blastocyst (expressed as increasing numerical score) and both the quality of the inner cell mass and the quality of the trophectoderm (grades A to C each). It should be stressed that a minority (*n* = 8) of the embryos were vitrified at morula stage (day 4). In these cases, blastocysts were warmed the day prior to the scheduled FRET so that at the end all transfers in the study (groups A and B) were done at blastocyst stage (day 5). Only blastocysts of qualities AA, AB, BA, and BB were taken into consideration for fresh and frozen transfer [6]. This holds also true for three cases (0,45%) of biopsied blastocysts included.

Patients receiving FRETs were stimulated with standard protocols, including the antagonist protocol, and long and ultralong protocols. The ovaries were stimulated using either recombinant FSH or human menopause gonadotropin. GnRH antagonists were used in the antagonist protocol, whereas GnRH agonists were used in the long and ultralong protocols. Thirty-six hours after ovulation induction using recombinant hCG, the oocytes were retrieved and IVF or ICSI was performed. In the case of low and normal responders (counted oocytes < 10), 1.500–3.000 IU of recombinant choriogonadotropin was administered after egg collection for luteal support. Endometrium transformation was induced by vaginal administered progesterone, and the embryos were transferred 5 days after oocyte retrieval.

Patients receiving FETs were treated with standard protocols using either modified natural cycles (with recombinant hCG for ovulation induction) or artificial cycles. The latter were mostly used from 2011 to 2020. In artificial cycles, patients were administered oral or transdermal oestrogen (E2) followed by endometrial transformation using vaginal administered progesterone. The embryos were transferred 5 days after progesterone administration. In some cases, GnRH agonists have previously been used for hormone downregulation.

Fourteen days after the ET, serum hCG levels were estimated. Electrochemiluminescence analysis (ECLIA) of serum hCG was performed using COBAS 6000 from 2011 to 2016 and COBAS 8000 from 2016 to 2020 (Roche Holding AG, Basel, Switzerland). Only blood samples analyzed in our reference laboratory were accepted for the present study. The results were recorded as international units per millilitre (mU/ml). Data of all pregnancies with a serum hCG level ≥ 5 mU/ml were analysed. Transvaginal sonography was performed routinely 29 days after ET.

### Subgroups

1. Non-viable pregnancies: *Biochemical pregnancy* was defined as a pregnancy with elevated hCG concentration, but no evidence of a gestational sac on a transvaginal ultrasonographic examination. *Extrauterine pregnancy* was defined as an ectopic pregnancy with implantation of the embryo outside the uterine cavity. *Early miscarriage* was defined as an ultrasonographic diagnosis of intrauterine pregnancy in the absence of cardiac activity.
2. Viable pregnancies: *Late miscarriage* was defined as the occurrence of a miscarriage after ultrasonographic confirmation of cardiac activity until 24 weeks of gestation. *Live birth* was defined as delivery of a live-born neonate at ≥ 24 weeks of gestation. *Stillbirth* was defined as delivery of a dead neonate at ≥ 24 weeks of gestation.
3. Clinical pregnancies included viable pregnancies and early miscarriages.

### Outcome measures

The primary endpoint of this study was the comparison between the serum hCG values obtained 14 days after FRET and FET. All patients and subgroups were analysed. The secondary endpoint was determination of the hCG cut-off values with high sensitivity and specificity for predicting viable and clinical pregnancies and performing regression analyses of the independent covariates for hCG values.

All patients received a sFRET or sFET at the IVF clinic at the Johannes Kepler University Linz between 2011 and 2020. The embryos were transferred 5 days after IVF/ICSI in the FRET group and 5 days after progesterone administration in the FET group. Only blastocysts from day 5 were included. We excluded all patients with transfer of > 1 embryo, resulting multiple pregnancies after sET (monozygotic pregnancies), and patients in whom serum hCG levels were not measured 14 days after ET.

### Statistical analysis

Q-Q plots were used to test the normal distribution of the metric data. Normally distributed data were assessed using Student’s *t*-test, and non-normally distributed data were assessed using the Mann–Whitney *U* test. Categorical data were analysed using chi-square test, and continuous variables were analysed using the Wilcoxon rank-sum test. Receiver operating characteristic (ROC) curves were generated for FRET and FET to determine the hCG cut-off values with the optimal sensitivity and specificity for identifying viable and clinical pregnancies. A multivariable regression model was used to assess potential covariates influencing the hCG concentrations. Owing to multiple transfers, ANOVA was performed to analyse whether these patients had a significant influence on hCG levels. SPSS (version 23.0; IBM, Armonk, NY, USA) was used for all statistical analyses and a *p*-value lower than 0.05 was considered as statistically significant. This study was approved by the local ethics committee (No: 1164/2021; 18 August 2021). Due to the retrospective nature of this study, informed consent was not obtained.

## Results

**Fig. 1:**
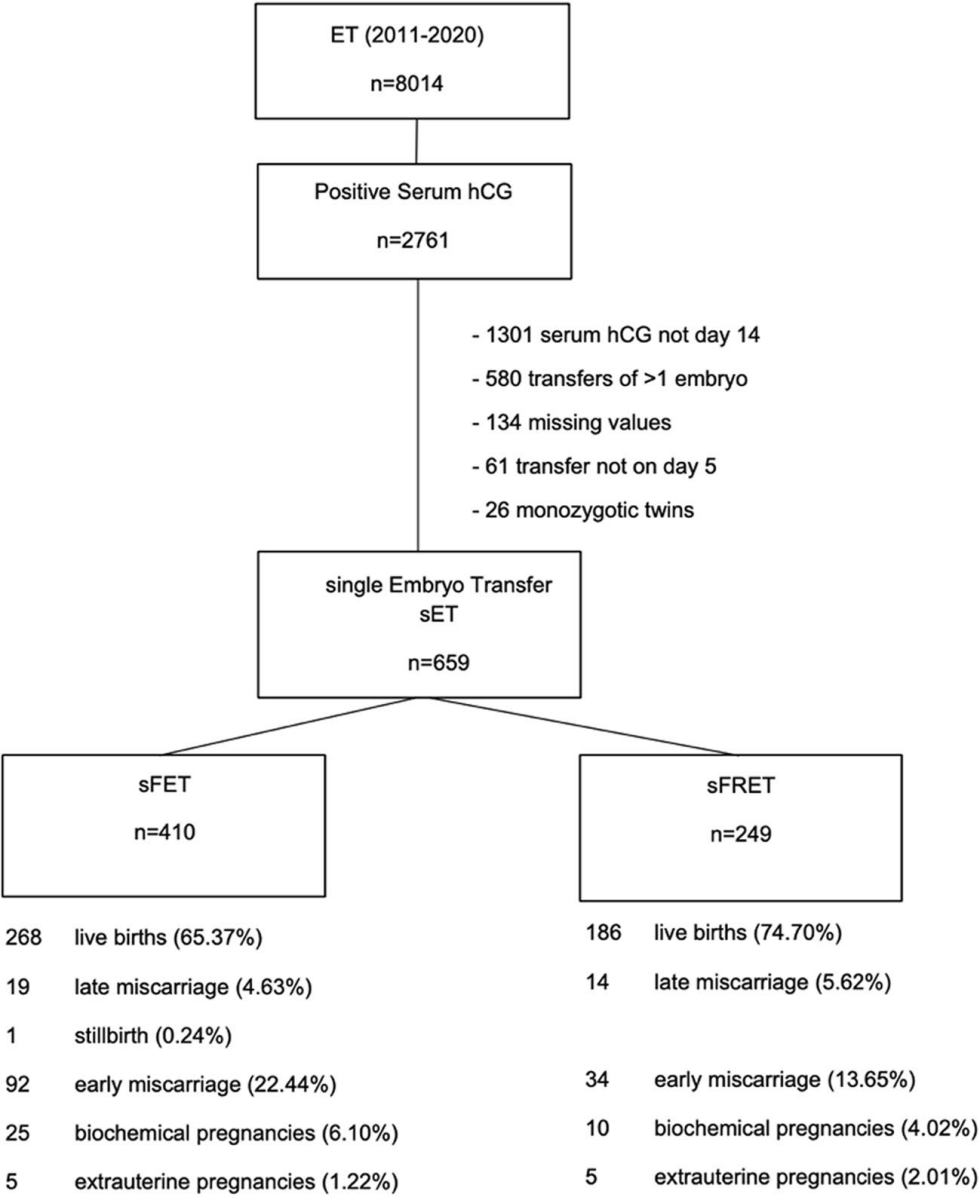
Flowchart of the patients included ET, embryo transfer; hCG, human chorionic gonadotropin; SET, single embryotransfer; sFET, single frozen embryo transfer; sFRET, single fresh embryo transfer

A total of 8,014 ETs were performed from January 2011 to December 2020. Of these patients, 2761 showed a positive serum hCG result (pregnancy rate = 34.45%). The number of patients (*n* = 659) who met the inclusion criteria were 410 in the sFET group and 249 in the sFRET group. In those receiving FET, 288 transfers resulted in a viable pregnancy (268 live births, 19 late miscarriages, and one stillbirth). In those receiving FRET, 200 transfers resulted in a viable pregnancy (186 live births and 14 late miscarriages). The number of transfers resulting in clinical pregnancies were 380 and 234 in the sFET and sFRET groups, respectively (Fig. 1).

The mean patient age was 31.6 ± 4.4 and 32.2 ± 4.3 years in the FRET and FET groups, respectively; no significant difference was observed (*p* = 0.111). Similarly, gestational age, mode of delivery, and the neonate’s sex in all the resulting live births were equal in both groups. Significantly higher birth weights were observed in the FET group than in the FRET group (3470.1 ± 572.6 g vs. 3137.4 ± 671.8 g; *p* < 0.001) (Table 1).

**Table 1:** Baseline characteristics

Variables (mean ± SD)		FRET (*n* = 249)	FET (*N* = 410)	*P* value
Maternal age (years)		31.6 ± 4.4	32.2 ± 4.3	**0.111**
Day of transfer (days)		5 ± 0	5 ± 0	**NS**
Day of hCG (days)		14 ± 0	14 ± 0	**NS**
Gestational age (weeks)		38.9 ± 2.8	39.4 ± 2.0	**0.109**
Birth weight (gram)		3137.4 ± 671.8	3470.1 ± 572.6	** < 0.001**
Mode of delivery	spontaneous	100	127	**0.562**
	assisted vaginal delivery	16	23	
	Caesarean section	67	106	
Sex	male	101	123	**0.175**
	female	82	130	
Mode of fertilization^a^	ICSI	225	200	**0.022**
	IVF	9	4	
	TESE	15	3	

*FRET* fresh embryo transfer, *FET* frozen embryo transfer, *NS* not significant, *hCG* human chorionic gonadotropin, *ICSI* intracytoplasmatic sperm injection, *IVF* in vitro fertilization, *TESE* testicular sperm extraction

^a^203 datapoints were missing in FET (data only available from 2015 to 2020)

Significantly higher hCG levels were found in those who received FET than in those who received FRET (1222.8 ± 946.7 mU/ml vs. 862.7 ± 572.9 mU/ml; *p* < 0.001) in all patients, in those who delivered live neonates (1521.5 ± 918.2 mU/ml vs. 1012.9 ± 555.4 mU/ml; *p* < 0.001), in those who achieved clinical pregnancies (1308.2 ± 930.0 mU/ml vs. 907.7 ± 560.8 mU/ml; *p* < 0.001) and in those who achieved viable pregnancies (1487.4 ± 917.2 mU/ml vs. 993.7 ± 548.7 mU/ml; *p* < 0.001). No difference in hCG values was observed in biochemical and extrauterine pregnancies, and early and late miscarriages. There was only one case of stillbirth in the FET group with an hCG value of 1232.0 mU/ml. Subgroup analysis showed higher median hCG values for FET in every subgroup, except for biochemical pregnancies, which had also a higher mean hCG value for FET (Table 2).

**Table 2:** Comparison of hCG values, total and subgroups

Groups		Fresh embryo transfer (*n* = 249)	Frozen embryo transfer (*n* = 410)	*P* Value
Total	number	249	410	**< 0.001**
	median (IQR)	793.6 (437.2–1145.0)	1100.0 (483–1800.0)	
	mean	862.7 ± 572.9	1222.8 mU/ml ± 946.7	
	range	10—2870	6—8298	
Biochemical pregnancy	number	10	25	**0.855**
	median (IQR)	53.0 (25.7–88.0)	55.0 (38.0–85.0)	
	mean	103.9 ± 140.3	79.0 ± 70.2	
	range	10—463	6—320	
Extrauterin pregnancy	number	5	5	**0.206**
	median (IQR)	264.0 (250.6–264.0)	375.0 (338.0–601.0)	
	mean	275.1 ± 53.2	450.8 ± 219.0	
	range	230—367	196—744	
Early miscarriage	number	34	92	**0.0501**
	median (IQR)	302.0 (166.6–567.0)	528.0 (192.6–1051.0)	
	mean	401.5 ± 309.8	747.0 ± 727.3	
	range	25.4—1155	16—3251	
Live birth	number	186	268	** < 0.001**
	median (IQR)	931.5 (575.0–1258.0)	1415.5 (880.5–2005.0)	
	mean	1012.9 ± 555.4	1521.5 ± 918.2	
	range	149—2870	100—8298	
Late miscarriage	number	14	19	**0.675**
	median (IQR)	612.5 (424.0–1047.0)	906.6 (402–1914)	
	mean	738.1 ± 378.2	1019.9 ± 809.3	
	range	319—1529	52.4—2796	
Stillbirth	number	-	1	**-**
	median (IQR)	-	1232.0	
	range	-	-	
Clinical pregnancy	number	234	380	** < 0.001**
	median (IQR)	849.0 (471.1–1155.0)	1176 (604.6–1869.5)	
	mean	907.7 ± 560.8	1308.2 ± 930.0	
	range	25.4—2870	16—8298	
Viable pregnancy	number	200	288	** < 0.001**
	median (IQR)	918.0 (566.5–1242.0)	1396.5 (864.5–1982.0)	
	mean	993.7 ± 548.7	1487.4 ± 917.2	
	range	149—2870	52.4—8298	

*IQR* interquartile range all hCG values presented in mU/ml the dashes denotes no data

**Fig. 2:**
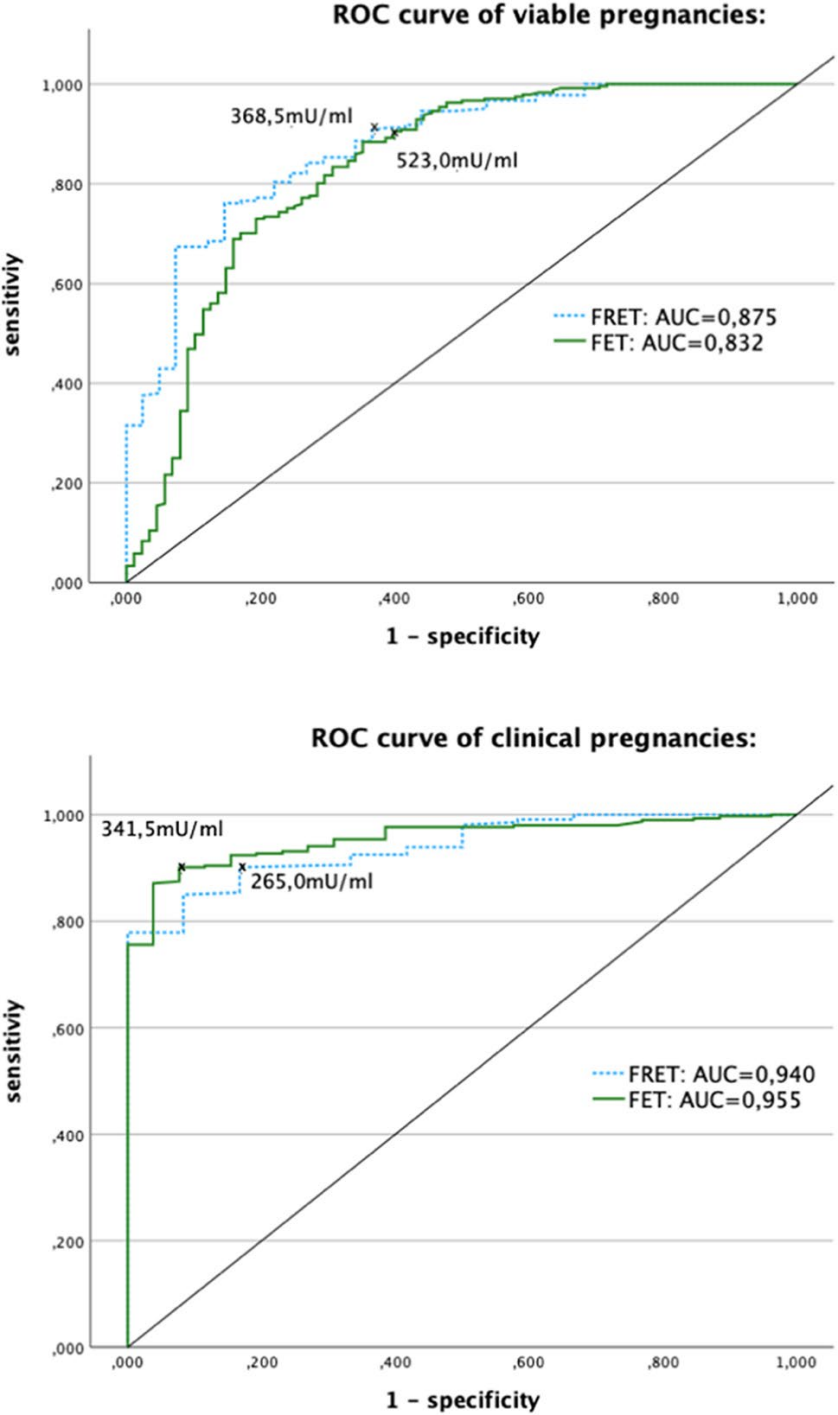
Receiver operating characteristic (ROC) curves for hCG levels in fresh embryo transfers (FRET- dotted blue line) and frozen embryo transfers (FET- continuous green line). Top image (viable pregnancy): threshold values predicting a viable pregnancy were 368.5 mU/ml (sensitivity 90.8%; specificity 63.4%) in FRET and 523.0 mU/ml in FET (sensitivity 90.5%; specificity 60.2%). Bottom image (clinical pregnancy): Threshold values predicting a clinical pregnancy were 265.0 mU/ml (sensitivity 90.1%; specificity 83.3%) in FRET and 341.5 mU/ml in FET (sensitivity 90.1%; specificity 92.3%)

The optimal hCG threshold values in viable pregnancies were 368.5 mU/ml (sensitivity 90.1%; specificity 63.4%) in FRET and 523.0 mU/ml (sensitivity 90.5%; specificity 60.2%) in FET. The area under the curve (AUC) for viable pregnancies was 0.875 (95% CI, 0.817–0.933) in FRET and 0.832 (95% CI, 0.776–0.888) in FET. The threshold hCG values in clinical pregnancies were 265.0 mU/ml (sensitivity 90.1%; specificity 83.3%) in FRET and 341.5 mU/ml (sensitivity 90.1%; specificity 92.3%) in FET. The AUC for clinical pregnancies was 0.940 (95% CI, 0.896–0.983) in FRET and 0.955 (95% CI, 0.929–0.980) in FET (Fig. 2).

Multivariable regression analyses revealed that the following variables did not affect maternal serum hCG levels: mode of fertilisation, endometrial thickness, and maternal age. HCG values were strongly affected when FET was performed. (Supplementary Table S1).

Multivariable regression analysis of FRET showed that anti Müllerian hormone (AMH) was a potential influencer of hCG levels. However, the medications used for stimulation (FSH or hMG), stimulation protocols, maternal age, and counted oocytes did not affect hCG (Supplementary Table S2).

In FET, one hCG value was an outlier, with a level of 8.298 mU/ml. Owing to this high runaway value, this case was double-checked for accuracy. A molar pregnancy or vanishing twin was excluded via an early transvaginal sonography; a healthy 2.910 g girl was born in the 40th week of gestation.

## Discussion

Till date, a single hCG measurement has not been used for the most likely prediction of viability and determining the pregnancy location. However, if single hCG values are available, they can provide clinicians with valuable information about the potential status and trends of pregnancy. Herein, we measured the serum hCG levels 14 days after FRET and FET to determine the differences between these two groups and their impact on pregnancy outcomes.

This study showed that hCG measurements were significantly higher in FET than in FRET in all patients, those with viable and clinical pregnancies, and those who delivered live neonates. Although several studies have evaluated the differences between FRET and FET, they included transfers of > 1 embryo [7–10]. The resultant multiple pregnancies and vanishing twins are associated with higher hCG values; therefore, the results from these studies should be interpreted cautiously. One study investigated the differences in hCG measurements between sFRET and sFET [11]. However, the hCG levels were measured on the 11th day after embryo transfer in this study. Although higher hCG levels were reported in FET than in FRET in the clinical pregnancies, there was no overall difference [11]. One explanation for this could be that differences in hCG levels on day 14 may be better identified owing to the naturally higher hCG values compared to than on day 11. Another study showed higher median hCG levels in sFET than in sFRET, where singleton live births occurred [12]; hCG was measured on day 19 of the luteal phase (equivalent to 14 days after IVF/ICSI and transfer of a day 5 embryo) but included both, cleavage stage with transfer on day 3 as well as blastocyst stage (day 5 and 6 blastocysts) transferred on day 5 after IVF/ICSI. In addition, blastocysts were transferred significantly more often in the FET group than in the FRET group in this study, and recombinant hCG was administered serially for up to 4 days before blood levels were sampled for hCG levels. A study by Zhu et al. also showed higher hCG levels in sFET than in sFRET on day 9 and 16 in viable pregnancies with gestational age > 13 weeks. In faster-growing blastocysts, higher hCG concentrations were observed on day 9 in sFRET, whereas no difference was observed on day 16 in sFRET and on days 9 and 16 in sFET [13].

During the window of implantation, E2 plays a major role in the process of embryo nidation into the receptive endometrium. Uterine glands are stimulated by nidatory E2 to secrete leukaemia inhibitory factor (LIF) which enables embryo adherence to the luminal epithelium. Uterine stromal cells differentiate into decidual cells and synthesise E2 which makes the uterus refractory to conceive and closes the window of implantation. Expression levels of the fibroblast growth factor subtypes during cell proliferation in the endometrial luteal phase differ with high- and low-dose E2 therapy [14]. E2 levels are reportedly higher in FRET than in FET due to ovarian hyperstimulation. These supraphysiologic E2 levels and premature progesterone elevation may affect the implantation window and embryo-endometrial synchronisation by slowing down the implantation process [12, 13]. These differences may be the cause for the different hCG levels in early pregnancy after MAR, higher hCG values in FET, and higher birth weights in FET [15]. Finally, high responders after IVF/ICSI with high E2 levels have lower cumulative live birth rates and profit from a “freeze all” strategy [16].

To date, no existing data on the optimal time point for serum hCG measurements after IVF are available. Currently, initial hCG levels after ET have been measured over a wide range: 9 days [13, 17], 10 days [18], 11 days [11], 12 days [8], 13 days [19, 20], 14 days [21], 15 days [22], 16 days [13], and 17 days [7]. After 9 days, pregnancies with “lower than excepted” hCG values after FRET and with slow-growing characteristics do not always have a poorer prognosis and can have ‘normal’ hCG values one week later [13]. Furthermore, hCG and recombinant hCG alfa are commonly used to induce ovulation in both FRET and FET (MNC); serum hCG levels increase immediately after the injection. Due to the half-life value of hCG (30–37 h) and depending on the initial administered dosage, serum hCG levels can be elevated up to 14 days after exogenous hCG administration [23]. The authors of this study highlighted, that too early performed quantitative pregnancy tests may be associated with false- positive results and should be interpreted cautiously.

Additionally, there are also no existing recommendations regarding the number of hCG blood samples to be obtained after IVF. Some clinicians measure hCG levels once, while others serially measure hCG levels at intervals ranging from 48 h [18, 22] to 168 h [13, 17]. It should be kept in mind that, in principle, the fewer hCG measurements are done the higher would be the risk to miss early miscarriages and in particular ectopic pregnancies. For this reason, working with single hCG values always requires ultrasound examinations and careful inquiry of clinical symptoms. The combined approach of hCG value and ultrasound is generally accepted [11, 12] and standard care in our tertiary referral centre.

In this study, we determined the threshold hCG values in viable and clinical pregnancies; higher hCG thresholds were observed in viable pregnancies. This could be explained by the fact that early miscarriages were classified as clinical pregnancies; miscarriages have naturally lower hCG values due to their disturbed vitality. This inclusion of early miscarriages as clinical pregnancies also explains the higher specificities in clinical pregnancies and the differences between the two ROC curves with a greater distance from the midline seen in clinical pregnancies (Fig. 2). The calculated threshold hCG values can be used to optimize care in patients undergoing IVF. Therefore, higher hCG values in FET should be considered.

According to the divergent findings in hCG levels analyzed on day 11 after blastocyst transfer [11] compared to day 14 in this study, we hypothesized that day 11 may be too early to determine a difference in hCG levels and the fact that too early taken blood samples may be influenced by exogenous hCG [23], together with the findings of this study with high sensitivities (90.1% in FRET and 90.5% in FET) determining a viable pregnancy, it is indicated that a single day 14 serum hCG value indeed might be sufficient for monitoring pregnancies resulting from MAR.

Multivariable regression analysis showed a potential correlation between “hCG levels” and “mode of transfer” when FET was performed. This was expected because of the higher hCG values observed in the different subgroups and in all patients. Additionally, our findings show that AMH potentially affected the hCG levels. Qui et al. showed that older adults undergoing IVF/ICSI had a negative correlation with serum hCG levels when FET was performed [18]. They suggest determining different cut off values for different age groups due to this. Younger patients reportedly have higher natural AMH levels; thus, the link between AMH as an effector on hCG levels was expected.

The restricted “number of transferred embryos” at our IVF clinic and the resultant high number of sETs performed over the last decade are strengths of this study. Furthermore, the fact that blastocysts of comparable quality (BB or better) were transferred in FRET and FET minimized the potential influence of embryo quality on hCG value and implantation.

The limitations of this study are its retrospective design and the high number of patients who were excluded because the blood samples were not obtained on day 14 (*n* = 1301). Blood sample collection was normally scheduled 14 days after IVF/ICSI. However, if day 14 fell on a Saturday or Sunday, the blood samples were collected on the next working day, which was 15 or 16 days after IVF/ICSI. There was also a certain overlap of hCG values amongst the different subgroups (e.g. viable and non-viable pregnancies) which highlights the importance of performing time-shifted transvaginal ultrasound in all patients when using single hCG levels for pregnancy monitoring. Certainly, exclusive reliance on single hCG values bears the risk to miss extrauterine pregnancies. In case of clinical symptoms (abdominal pain., bleeding), immediate gynecological examination for the early diagnosis of ectopic pregnancy is indispensable.

In conclusion, significantly higher serum hCG values were found in the sFET group than in the sFRET group on day 14 after ET. Higher hCG values do not imply higher pregnancy or live birth rates; however, they may be linked to higher birth weights in infants. It is suggested that a single serum hCG value (in combination with obligate subsequent ultrasound examination to exclude ectopic pregnancies) could be used for monitoring pregnancies resulting from IVF/ICSI.

### Supplementary Information

Below is the link to the electronic supplementary material.

Supplementary file1 (DOCX 14 KB)

Supplementary file2 (DOCX 14 KB)

## Data Availability

The data that support the findings of this study are available upon reasonable request.
