# Supplementary material for: Single day 14 serum hCG values allow prediction of viable pregnancy and are significantly higher in frozen as compared to fresh single blastocyst transfer
Source: J Assist Reprod Genet. 2024 Jun 13;41(8):2193–200. doi: 10.1007/s10815-024-03164-z (PMC11339198; doi:10.1007/s10815-024-03164-z)
Supplement: Supplementary file 1 — Supplementary file1 (DOCX 14 KB) [file 10815_2024_3164_MOESM1_ESM.docx]

**supplementary table S1** multivariable regression analysis determining possible associations between co- factors and serum hCG values in FRET and FET

| **Variables** | **Regression- coefficient** | **95% CI** | **P Value** |
| --- | --- | --- | --- |
| FET | 464.11 | 224.53 to 703.694 | <0.001 |
| ICSI | 545.91 | -254.20 to 1346.01 | 0.181 |
| IVF | 681.62 | -345.42 to 1708.66 | 0.193 |
| Endometrium thickness | -5.99 | -69.58 to 57.59 | 0.853 |
| Maternal age | -2.51 | -28.42 to 23.93 | 0.849 |
| FRET- fresh embryo transfer; FET- frozen embryo trasnfer; ICSI- intracytoplasmatic sperm injection; IVF- in vitro fertilization | | | |
