# Supplementary material for: Single day 14 serum hCG values allow prediction of viable pregnancy and are significantly higher in frozen as compared to fresh single blastocyst transfer
Source: J Assist Reprod Genet. 2024 Jun 13;41(8):2193–200. doi: 10.1007/s10815-024-03164-z (PMC11339198; doi:10.1007/s10815-024-03164-z)
Supplement: Supplementary file 2 — Supplementary file2 (DOCX 14 KB) [file 10815_2024_3164_MOESM2_ESM.docx]

**supplementary table S2** multivariable regression analysis determining possible associations between co- factors and serum hCG values in FRET

| **Variables** | **Regression- coefficient** | **95% CI** | **P Value** |
| --- | --- | --- | --- |
| FSH | -185.46 | -477.52 to 106.61 | 0.213 |
| HMG | -226.56 | -518.13 to 65.00 | 0.128 |
| Antagonist protocoll | -193.57 | -401.06 to 13.92 | 0.067 |
| AMH | 35.58 | 11.53 to 59.64 | 0.004 |
| Maternal age | -15.84 | -35.30 to 3.63 | 0.111 |
| COC | 11.71 | -5.47 to 28.89 | 0.182 |
| FRET- fresh embryo transfer; FSH- follicle stimulating hormone; hMG- human menopause gonadotropin; AMH- anti Müllerian hormone; COC- counted oocytes | | | |
